# Pyrexia-Induced Brugada Syndrome Unmasked by Community-Acquired Pneumonia: A Case Report

**DOI:** 10.7759/cureus.89907

**Published:** 2025-08-12

**Authors:** Homodi Mohamed, Ahmed Elnour, Mohamed Karar, Mohamed Omer, Hatim A Yagoub

**Affiliations:** 1 General Practice, University Hospital Kerry, Kerry, IRL; 2 Internal Medicine, University Hospital Kerry, Kerry, IRL; 3 Acute Medicine, Letterkenny University Hospital, Letterkenny, IRL; 4 Internal Medicine, University Hospital Waterford, Waterford, IRL; 5 Cardiology, University Hospital Kerry, Kerry, IRL

**Keywords:** arrhythmia, brugada syndrome and fever, community-acquired pneumonia (cap), pyrexia, ventricular tachycardia (vt)

## Abstract

Brugada syndrome is a genetic arrhythmic disorder marked by electrocardiographic abnormalities and a heightened risk of sudden cardiac death, commonly unmasked by various clinical triggers. Although drug exposure and electrolyte disturbances are well-recognized precipitants, fever is an important but underappreciated factor capable of revealing a latent Brugada pattern and provoking malignant ventricular arrhythmias. We present the case of a previously healthy 34-year-old man who developed sustained ventricular tachycardia (VT) following the onset of fever secondary to community-acquired pneumonia. His initial presentation included high-grade fever, productive cough, and pleuritic chest pain. Electrocardiography revealed a type 1 Brugada pattern coinciding with his febrile state, along with frequent ventricular ectopy. The patient subsequently experienced an episode of sustained VT, which required acute stabilization. Following recovery, he underwent placement of an implantable cardioverter-defibrillator for secondary prevention of sudden cardiac death. The case underscores the potential for fever to serve as a trigger for Brugada syndrome and highlights the importance of recognizing this association in febrile patients presenting with suggestive electrocardiographic changes. Prompt identification and management, including aggressive fever control and appropriate implementation of device therapy, are critical for preventing life-threatening outcomes in individuals predisposed to Brugada syndrome. This report aims to raise awareness among clinicians about pyrexia-induced unmasking of Brugada syndrome and emphasizes the necessity for vigilant monitoring in similar high-risk situations.

## Introduction

Brugada syndrome is an inherited cardiac channelopathy notable for its signature electrocardiographic patterns and an increased risk of sudden cardiac death in affected individuals [1,2]. Although the condition is commonly associated with genetic mutations affecting sodium channel function, its clinical manifestation often depends on the presence of external triggers [3]. Fever is an increasingly recognized but underappreciated factor capable of unmasking a latent Brugada phenotype, particularly in otherwise asymptomatic individuals [1,4,5]. In the context of an acute infectious process, such as community-acquired pneumonia, febrile episodes can precipitate malignant ventricular arrhythmias, posing significant risks if not promptly identified and managed [1-5]. This case highlights the critical relationship between pyrexia and Brugada syndrome, underscoring the importance of vigilance in evaluating febrile patients who present with concerning electrocardiographic changes [1-4].

## Case presentation

A 34-year-old man of Irish ethnicity presented to the emergency department with a three-day history of fever, productive cough, and left-sided pleuritic chest pain, accompanied by profuse diaphoresis and nausea. He denied any prior episodes of syncope or palpitations. He was a heavy smoker, consumed alcohol, and reported symptoms suggestive of nocturnal apnea. His medical and family histories were unremarkable for cardiovascular disease or sudden cardiac death. Employed as a headstone erector, he remained a current smoker and drinker.

**Figure 1:**
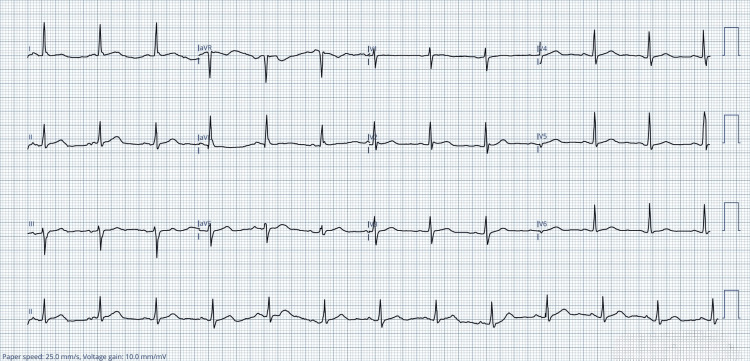
The electrocardiogram obtained at the triage demonstrated a normal sinus rhythm aVR: augmented vector right; aVL: augmented vector left; aVF: augmented vector foot

On examination, his body mass index was 28 kg/m². He was febrile with a temperature of 38.5°C, had a heart rate of 73 beats/minute, blood pressure of 120/58 mmHg, and a respiratory rate of 26 breaths/minute. Chest auscultation revealed reduced air entry with crackles predominantly in the left lower lung zone. Chest radiography demonstrated a patchy consolidation within the left lower lobe consistent with pneumonia. Laboratory investigations demonstrated leukocytosis, with a white blood cell count of 12.8 × 10⁹/L, hemoglobin level of 14.2 g/dL, and thrombocytosis with a platelet count of 504 × 10⁹/L. C-reactive protein was markedly elevated at 166 mg/L. The initial troponin level obtained at triage was less than 3, and the electrocardiogram (ECG) (Figure 1) demonstrated normal sinus rhythm (NSR). Renal function, serum magnesium, and liver function tests were all within normal limits. Viral screening was negative for COVID-19 but tested positive for influenza B.

**Figure 2:**
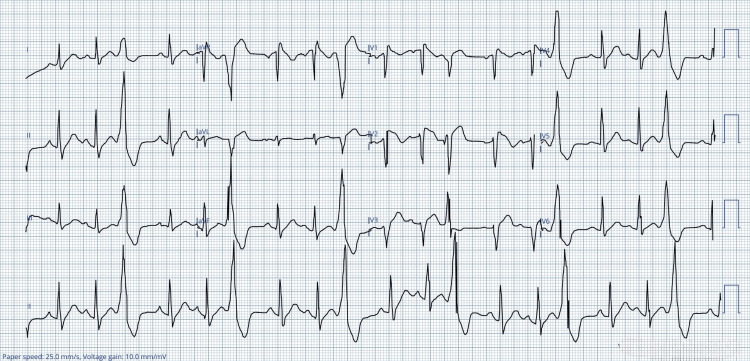
Repeated ECG showing multiple premature ventricular contractions in a trigeminy pattern and coved ST-segment elevation in leads V1-V2 aVR: augmented vector right; aVL: augmented vector left; aVF: augmented vector foot; ECG: electrocardiogram

A repeat ECG (Figure 2) was performed an hour later due to worsening pleuritic chest pain. It demonstrated multiple premature ventricular contractions in a trigeminy pattern and coved ST-segment elevation in leads V1-V2, findings consistent with Brugada syndrome.

**Figure 3:**
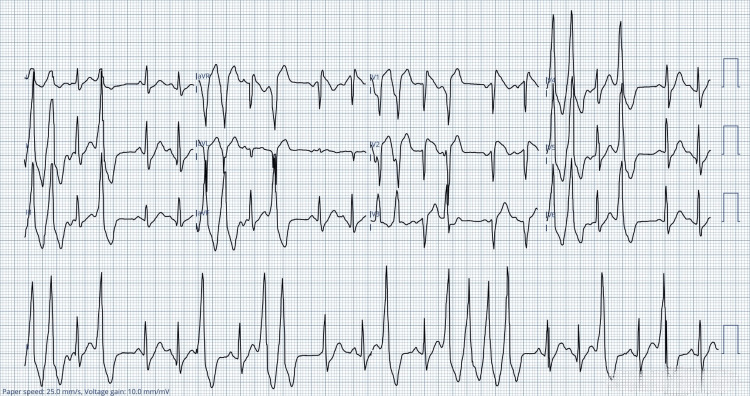
The third ECG showing nonsustained ventricular tachycardia with coved ST-segment elevation in leads V1-V2 aVR: augmented vector right; aVL: augmented vector left; aVF: augmented vector foot; ECG: electrocardiogram

While the patient was waiting in the emergency department, he experienced a sudden onset of strong palpitations. The third ECG (Figure 3) was done immediately and showed nonsustained ventricular tachycardia (VT) with coved ST-segment elevation in leads V1-V2.

**Figure 4:**
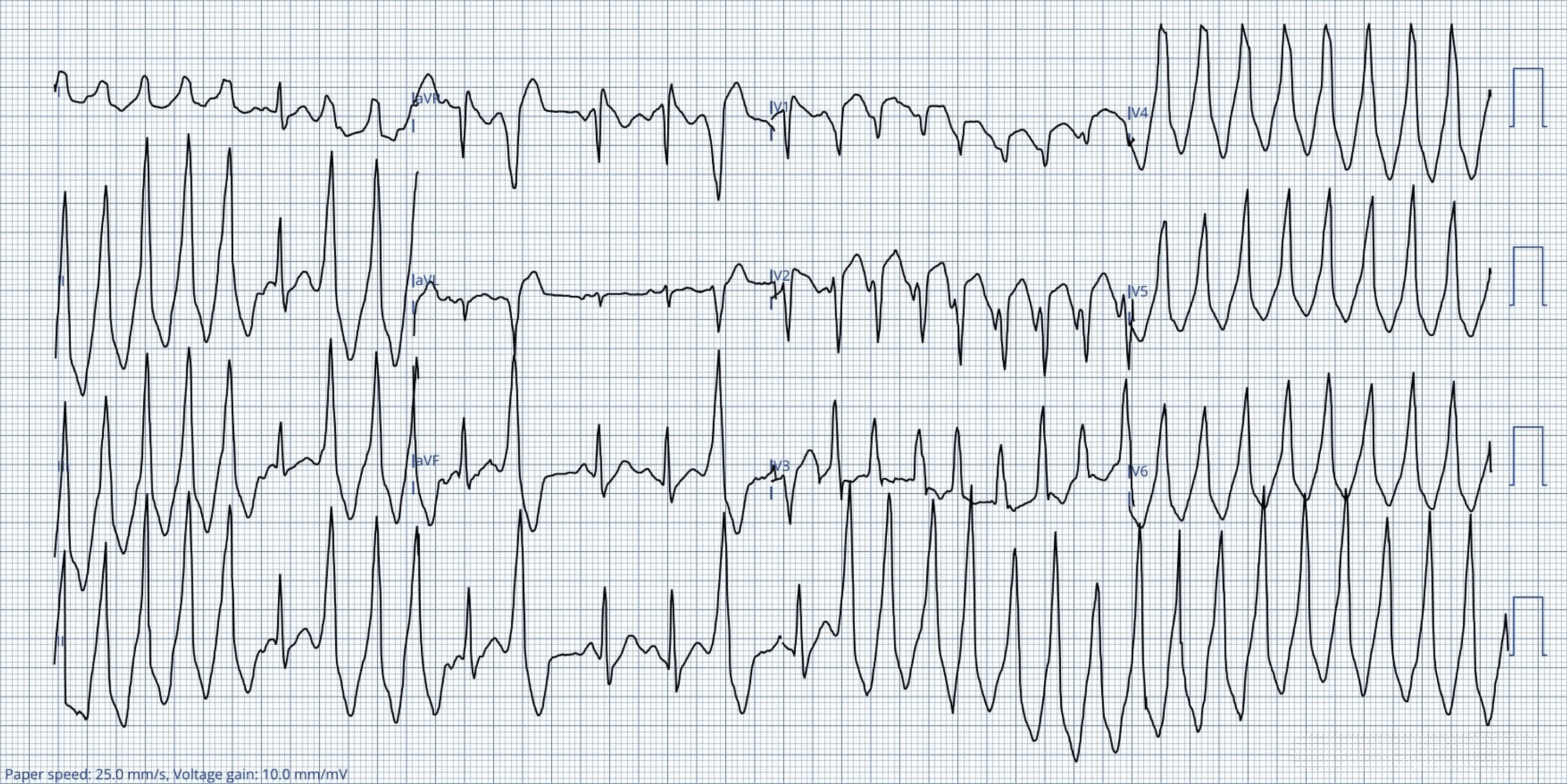
Fourth ECG: the cardiac monitor showing sustained ventricular tachycardia aVR: augmented vector right; aVL: augmented vector left; aVF: augmented vector foot; ECG: electrocardiogram

The patient was transferred to the resuscitation area, where continuous cardiac monitoring revealed sustained VT (Figure 4). He was initially managed as a case of ST-segment elevation myocardial infarction with VT and received intravenous paracetamol, amiodarone 300 mg, aspirin 300 mg, morphine 5 mg, ticagrelor 180 mg, and a stat dose of magnesium sulfate 2 g. Antibiotic therapy with Augmentin and clarithromycin was administered for pneumonia. Cardiological consultation established a diagnosis of pyrexia-induced Brugada syndrome, and the patient was referred to a tertiary center for specialized electrophysiological management. With resolution of his systemic symptoms, his cardiac rhythm normalized, though dynamic ST-segment changes were observed, worsening during febrile episodes. Ten days following admission, he became clinically stable and was deemed fit for implantable cardioverter defibrillator (ICD) insertion. Transthoracic echocardiography demonstrated normal cardiac function.

**Figure 5:**
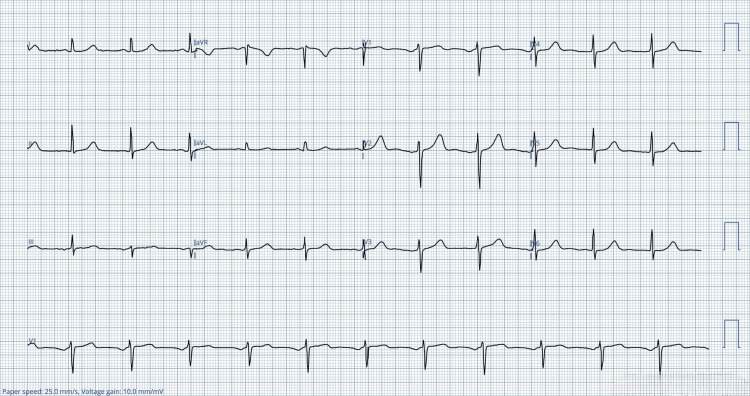
The postimplantation electrocardiogram demonstrated normal sinus rhythm aVR: augmented vector right; aVL: augmented vector left; aVF: augmented vector foot

The postimplantation ECG (Figure 5) demonstrated NSR. The patient was thereafter discharged home in a stable clinical condition.

He was referred to a respiratory physician to further evaluate for obstructive sleep apnea, and genetic testing was arranged. Outpatient follow-up was scheduled for ongoing ICD management. The patient received counseling regarding strict avoidance of alcohol and substances known to exacerbate Brugada syndrome, with reference to resources such as the Brugada Drugs website [6]. He was also advised by his general practitioner to aggressively manage any future febrile episodes using paracetamol and, if necessary, ibuprofen.

## Discussion

Brugada syndrome is a heritable cardiac channelopathy predominantly associated with sodium channel dysfunction, conferring a significant risk of life-threatening ventricular arrhythmias and sudden cardiac death, particularly in young and otherwise healthy individuals [5-7]. While the classic electrocardiographic pattern may be absent under baseline conditions, various external factors, most notably fever, can unmask or exacerbate the Brugada phenotype.

Fever is a well-documented precipitant of Brugada syndrome manifestations, owing to temperature-dependent impairment of sodium channel function in genetically susceptible individuals [5]. Even modest elevations in body temperature can provoke malignant ventricular arrhythmias, as seen in this case, supporting fever’s role as a key modulator of arrhythmogenic risk in Brugada syndrome. The described patient developed sustained VT in the context of pneumonia-induced pyrexia, emphasizing the need for high clinical suspicion and prompt recognition in febrile patients with suggestive ECG changes.

The management of pyrexia-induced Brugada syndrome entails rapid identification and correction of the triggering factor, fever, through aggressive antipyretic therapy, while simultaneously avoiding medications known to exacerbate Brugada syndrome (such as certain antiarrhythmics and psychotropics) [5-7]. In the acute setting, the priority is prevention of recurrent arrhythmias and reduction of sudden cardiac death risk. For patients who have survived a ventricular arrhythmic event, as in this report, definitive therapy with implantable cardioverter-defibrillator (ICD) placement is strongly indicated and is supported by prevailing guidelines [8].

This case also highlights the importance of comprehensive patient education regarding fever management, avoidance of contraindicated substances, and the significance of family screening where appropriate, given the genetic nature of Brugada syndrome. The outcome in this patient was favorable following ICD insertion and systematic follow-up, consistent with reports demonstrating improved survival with device therapy in symptomatic patients [8].

Clinicians must remain vigilant for Brugada syndrome in any patient presenting with new-onset arrhythmia and fever, even in the absence of prior cardiac symptoms or family history. Increased awareness can facilitate early diagnosis, allow implementation of preventive strategies, and ultimately reduce mortality associated with this potentially lethal but modifiable arrhythmogenic disorder [5,7,8].

## Conclusions

This case highlights the critical role of fever as a precipitating factor for arrhythmic events in patients with latent Brugada syndrome. Prompt recognition and management of pyrexia-induced Brugada syndrome-including aggressive fever control, avoidance of contraindicated medications, and early consideration of implantable cardioverter-defibrillator therapy-are essential to preventing life-threatening outcomes. Clinicians should maintain a high index of suspicion for Brugada syndrome in febrile patients presenting with suggestive ECG changes, as early intervention can significantly improve prognosis.
